# A Mouse with a Monoclonal Primary lmmunoglobulin Repertoire not Further Diversified by V-Gene Replacement

**DOI:** 10.1155/1999/24514

**Published:** 1999

**Authors:** Marilia Cascalho, Denise A. Martin, Jamie Wong, Queenie Lam, Matthias Wabl, Gillian E. Wu

**Affiliations:** 1 Department of Microbiology and Immunology University of California San Francisco CA 94143 USA; 2 Department of Immunology Ontario Cancer Institute University of Toronto Toronto Ontario M5G 2M9 Canada; 3 Ontario Cancer Institute Room 8-113 610 University Avenue Toronto Ontario M5G 2M9 Canada

**Keywords:** MonoB mouse, QM mouse, RAG, transgenic, knockin, flow cytometry

## Abstract

We have generated a monoclonal B-cell mouse by introducing homozygous, nonfunctional RAG-2 alleles and a *λ*1 light-chain transgene into the quasi-monoclonal (QM) mouse, which contains a “knocked-in” V_H_DJ_H_ rearrangement. Thus, this mouse, which we call MonoB, is devoid of T cells and contains preformed heavy- and light-chain genes encoding immunoglobulin with an anti-NP specificity. The MonoB mouse allows us to examine immunoglobulin diversity in the absence of processes mediated by V(D)J recombination and T cells. Here we report that not only is the MonoB's primary immunoglobulin repertoire monoclonal, but also that its secondary repertoire is not further diversified by V-gene replacement or gene conversion. Among 99 heavy-chain and 41 *λ* light-chain genes from peripheral B cells of the MonoB mouse, there were no V-gene replacements. When compared to the QM mouse, which has RAG activity, and for which V-gene replacement is the major diversifying mechanism, these data suggest that V-gene replacement is mediated by V(D)J recombination and not by other recombination systems.


*Developmental Immunology*, 1999, Vol. 7(1), pp. 43-50
Reprints available directly from the publisher
Photocopying permitted by license only

© 1999 OPA (Overseas Publishers Association)
N.V. Published by license under the
Harwood Academic Publishers imprint,
part of Gordon and Breach Publishing Group
Printed in Malaysia

### A Mouse with a Monoclonal Primary Immunoglobulin Repertoire not Further Diversified by V-Gene Replacement

MARILIA CASCALHO[a], DENISE A. MARTIN[b], JAMIE WONG[a], QUEENIE LAM[b], MATTHIAS WABL[a] and GILLIAN E. WU[b]*

[a]*Department of Microbiology and Immunology, University of California, San Francisco, CA 94143 and* [b]*Department of Immunology and Ontario Cancer Institute, University of Toronto, Toronto, Ontario M5G 2M9*

*(Revised 01 December, 1998; In final form 09 December, 1998)*

We have generated a monoclonal B-cell mouse by introducing homozygous, nonfunctional RAG-2 alleles and a λ1 light-chain transgene into the quasi-monoclonal (QM) mouse, which contains a "knocked-in" $V_HDJ_H$ rearrangement. Thus, this mouse, which we call MonoB, is devoid of T cells and contains preformed heavy- and light-chain genes encoding immunoglobulin with an anti-NP specificity. The MonoB mouse allows us to examine immunoglobulin diversity in the absence of processes mediated by V(D)J recombination and T cells. Here we report that not only is the MonoB's primary immunoglobulin repertoire monoclonal, but also that its secondary repertoire is not further diversified by V-gene replacement or gene conversion. Among 99 heavy-chain and 41 λ light-chain genes from peripheral B cells of the MonoB mouse, there were no V-gene replacements. When compared to the QM mouse, which has RAG activity, and for which V-gene replacement is the major diversifying mechanism, these data suggest that V-gene replacement is mediated by V(D)J recombination and not by other recombination systems.

*Keywords:* MonoB mouse, QM mouse, RAG, transgenic, knockout, knockin, flow cytometry

### INTRODUCTION

$V_H$ replacement, as originally described *in vitro* (Kleinfield et al., 1986; Reth et al., 1986; Beck-Engeser et al., 1987; Kleinfield and Weigert, 1989 ; Shirasawa et al., 1992) and in acute lymphoblastic leukemia (Steenbergen et al., 1993), can generate a heavy (H) chain with a different specificity (Kleinfield et al., 1986; Reth et al., 1986; Cascalho et al., 1996) or destroy a productive gene (Taki et al., 1995). $V_H$ replacement is thought to be mediated by a heptamer recombination signal sequence (RSS) embedded near the 3′ end of many $V_H$'s (Covey et al., 1990; Usuda et al., 1992: Chen et al., 1995; Fanning et al., 1998) and by a putative nonamer sequence found ca. 12 bp upstream in the transgene (Chen et al., 1995). Because V-gene replacement shares the same RSS as V(D)J recombination, it has been proposed that the two share the same mechanism (Covey et al., 1990;

* Corresponding authors: Ontario Cancer Institute, Room 8–113, 610 University Avenue, Toronto, Ontario M5G 2M9, Canada. Tel.: +1 416 946 2171. Fax: +1 416 946 2086. E-mail gillian.wu@utoronto.ca.

Usuda et al., 1992; Chen et al., 1995; Fanning et al., 1998).

In order to determine whether V-gene replacement is dependent on V(D)J recombinase activity, we bred a mouse with preformed IgH and IgL genes onto a RAG-knockout background. For the heavy- (H-) chain locus, we used the quasi-monoclonal (QM) mouse, which is heterozygous for two gene-targeted alleles: On one homologue is a targeted insertion in which the stretch of DNA containing the $J_H$ segments has been replaced by a rearranged $V_HDJ_H$ exon encoding an H-chain V region bearing the 17.2.25 idiotope (id) (Cascalho et al., 1996); on the other homologue is a nonfunctional "knockout" in which all $J_H$ gene segments have been deleted (Chen et al., 1993a). The $J_\kappa$ stretch has been knocked out on both homologues (Chen et al., 1993b), and, as a consequence, QM mice produce only λ-type light (L) chains. We bred the QM mouse to the RAG-2-deficient strain (Shinkai et al., 1992) to generate a mouse homozygous for a disrupted RAG-2 allele and containing the inserted 17.2.25 $V_HDJ_H$ exon. As the absence of RAG expression also prevents V to J rearrangement at the λ L-chain locus, we introduced a λ1 transgene (Young et al., 1994). The absence of RAG-2 expression also means that the resulting mouse lacks T cells. This mouse, which we call MonoB, has a nominally monoclonal primary B-cell repertoire with a B-cell receptor (BCR) consisting of an H chain encoded by an inserted $V_HDJ_H$ (with a constant region) and the λ1 transgene; this BCR is specific for the hapten NP [(4-hydroxy-3-nitrophenyl) acetyl] (Cascalho et al., 1996).

We report here experiments to investigate whether the secondary repertoire of the MonoB mouse becomes diversified by V-gene replacement, gene conversion, or any other process involving transfer of donor sequences.

### RESULTS

### Flow Cytometric Analysis

Figure 1 shows flow-cytometric profiles of peripheral blood lymphocytes (PBL) from MonoB, QM, and RAG-$2^{-/-}$ mice stained with an anti-idiotypic antibody (Cascalho et al., 1996) specific for the H chain encoded by the inserted 17.2.25 $V_HDJ_H$ and either anti-B220 (Figs. 1A-1C) or anti-$\mu^a$ (Figs. 1D-1F), Figure 2 shows flow-cytometric profiles of peripheral blood lymphocytes from MonoB, QM, and RAG-$2^{-/-}$ mice stained with an anti-λ chain antibody (Cascalho et al., 1996) and either anti-B220 (Figs. 2A and 2B) or anti-CD19 (Figs. 2C to 2E).

As can also be seen in Fig. 1, there are no apparent $\mu^a$-negative, id-positive (switched) cells in the MonoB mouse (Fig. 1D), although as expected, the QM mouse has switched cells (Fig. 1E). Analysis of serum Ig also failed to detect H-chain isotypes other than IgM (data not shown).

As shown in Fig. 1 and 2, well over 20% of the peripheral B cells in MonoB mice do not have the idiotypic BCR. About 30% of the B220-positive (Fig. 1A) as well as the $\mu^a$-positive (Fig. 1D) cells are id-negative; about 25% of the B220-positive (Fig. 2A) and CD19-positive (Fig. 2C) cells are λ-negative. In QM mice, about 15% of the B220-positive (Fig. 1B) and $\mu^a$-positive (Fig. 1E) cells are id-negative, and only a few (<3%) of the B220-positive (Fig. 2B) or CD19-positive (Fig. 2D) are λ-negative. As the number of B220-positive cells in the periphery of MonoB is about one-third that of QM mice, the frequencies given in Figs. 1 and 2 mean that there are more λ1-negative but about the same or fewer id-negative cells in the PBL of MonoB than in those of QM mice.

As shown in Fig. 3, 7.6% of MonoB and 1.4% of QM PBL were B220-positive, CD43-positive. These cells are immature B-lineage cells, and they stain only dimly for B220 in both strains; as they mature, they will lose CD43 and increase the amount of B220 on their surface. But the id-negative and λ-negative populations of Figs. 1 and 2 stain brightly with anti-B220, so they are not likely to be immature B lymphocytes that have not yet expressed their BCR.

### Immunoglobulin RNA and DNA Sequence Analysis of Id-Negative and λ-Negative Cells

Table I gives a summary of Ig cDNA and genomic DNA sequences cloned from sorted id-negative and

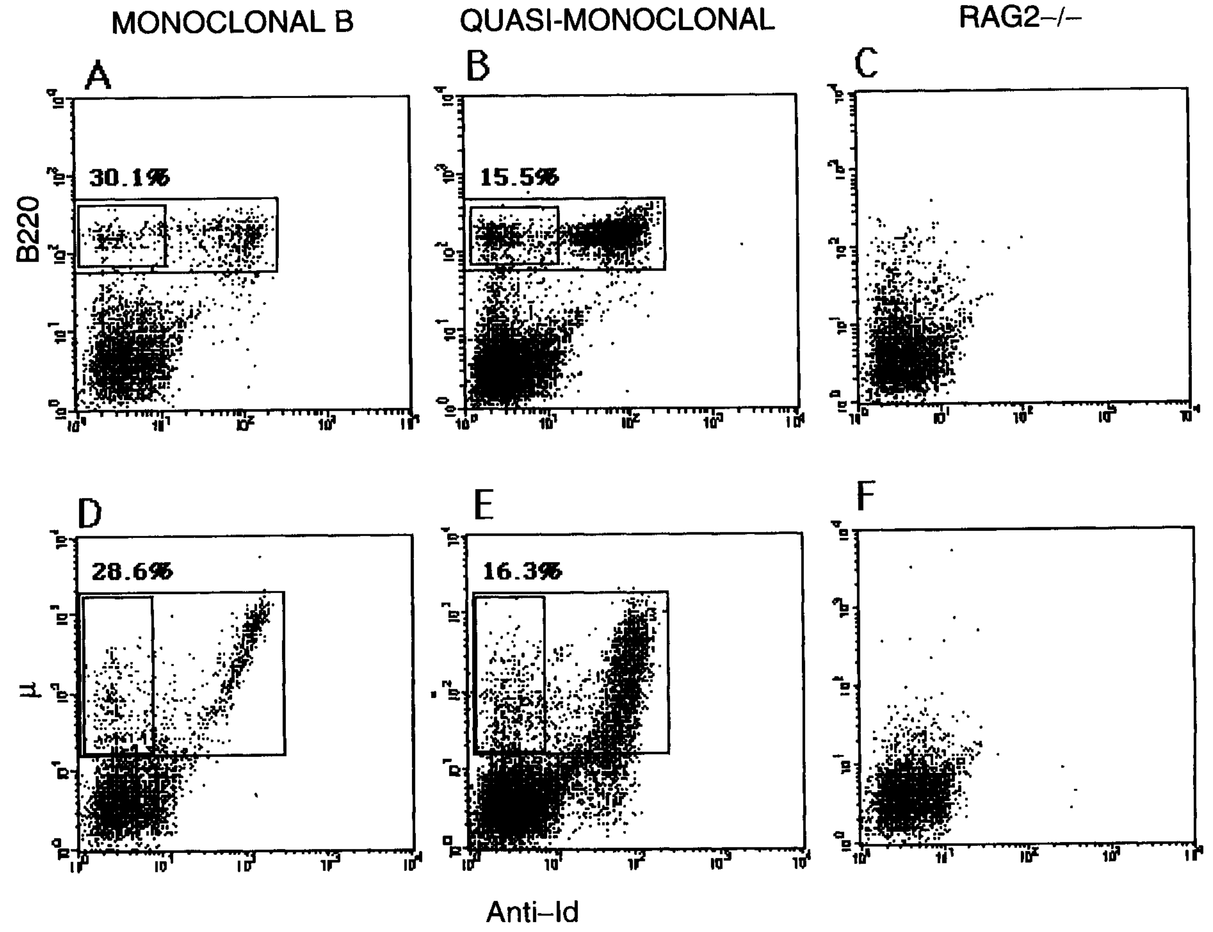

FIGURE 1 Flow-cytometric analysis of peripheral blood cells from MonoB (A, D), QM (B, E), and RAG-$2^{-/-}$ mice (C, F). *y* axis; PE-conjugated anti-B220 monoclonal antibody (A-C) or anti-$\mu^a$ (D-F) *x*-axis: FITC-conjugated anti-idiotype 17.2.25 (anti-id) monoclonal antibody from λ-negative cells as well as Ig cDNA sequences cloned from LPS-stimulated cells. All 140 sequences were clearly the 17.2.25 $V_HDJ_H$ or λ1, the knocked-in H-chain and transgenic L-chain genes present in the germline of MonoB mice, respectively. That is, there were no V-gene replacements. For the expressed λ-chain transgene, this is not surprising, for there are no likely donors for its replacement. But the frequency of V-gene replacements in the id-negative fraction of the QM mouse was previously found to be almost 100% (Cascalho et al., 1996).

There was also no evidence of gene conversion among the 140 variable-region gene segments sequenced. Moreover, in the same cDNA preparation and using the same reagents (except for the primers), there were more mutations in the $V_HDJ_H$ segment than in a similarly sized $C_\mu 1$–2 segment – 41 nucleotide changes in 6496 bases of the $V_HDJ_H$ segment vs. 5 nucleotide changes in 7935 bases of the $C_\mu 1$–2 segment. Nevertheless, because of the large number of PCR cycles used, an accurate assessment of the mutant frequency would require more extensive methodological controls.

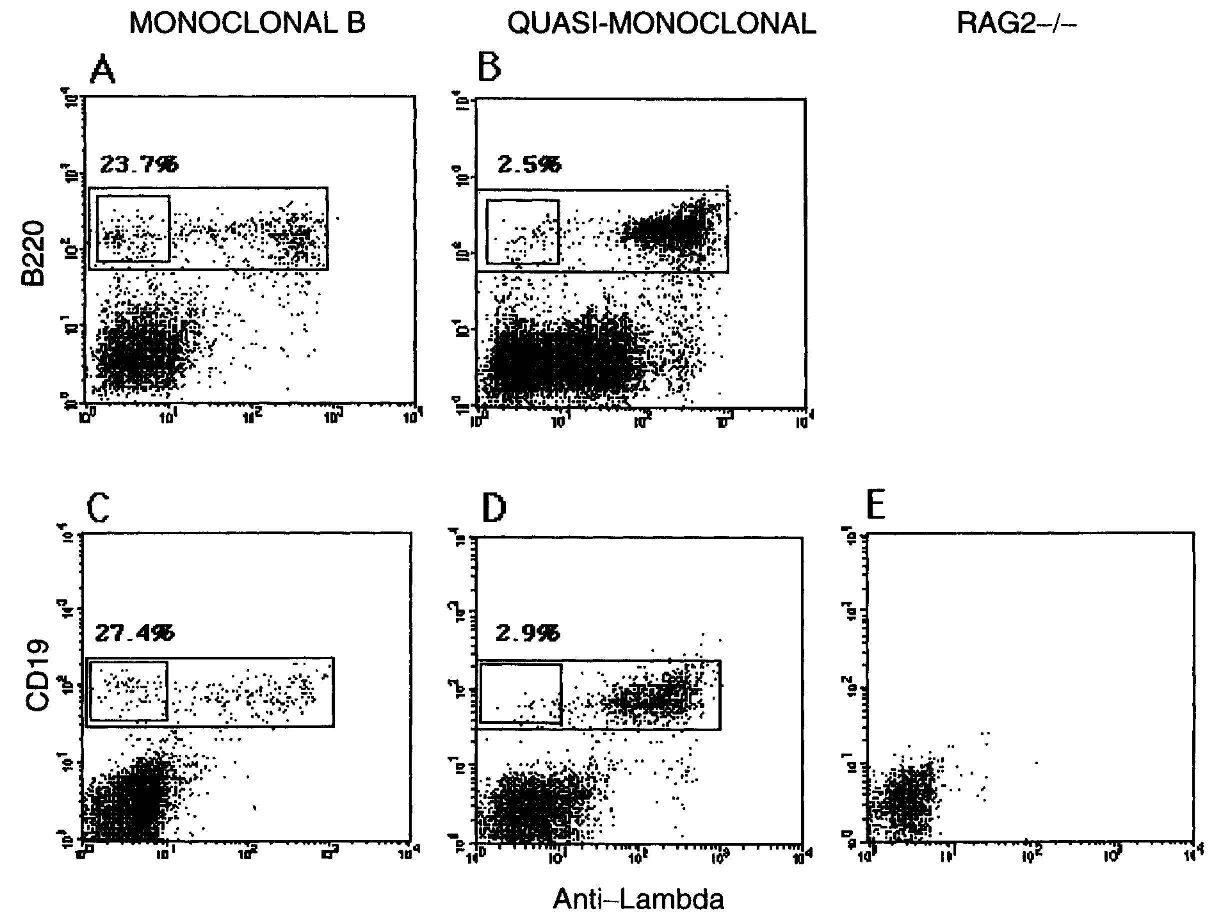

FIGURE 2 Flow-cytometric analysis of peripheral blood cells from MonoB (A, C), QM (B, D), and RAG-$2^{-/-}$ mice (E). *y* axis; PE-conjugated anti-B220 monoclonal antibody (A-B); or anti-CD19 (C-E). *x*-axis: FITC-conjugated anti-λ monoclonal antibody, which reacts with λ1 and λ2 L chains

### DISCUSSION

### What Are the Idiotype-Negative B Lymphocytes in MonoB Mice?

The high frequency of B-lineage cells lacking the idiotypic BCR of MonoB mice remains a puzzle. We have experimentally excluded that their lack of idiotype results from its loss by V-gene replacement, gene conversion, or any other process involving transfer of donor sequences. Moreover, we have experimentally excluded that these cells are immature B cells that have not yet expressed their BCR. Although we have not formally excluded the possibility that the lack of reaction with anti-idiotypic and anti-λ antibodies is due to somatic hypermutation, the mutation frequency is not high enough to make this a likely possibility unless the loss of idiotype per se confers a selective advantage.

It is possible that the relevant epitopes in these cells are masked by cross-reactive antigen(s), perhaps endogenous, perhaps environmental. Under normal circumstances, B cells with bound antigen home to the secondary lymphoid organs and, thus, are taken

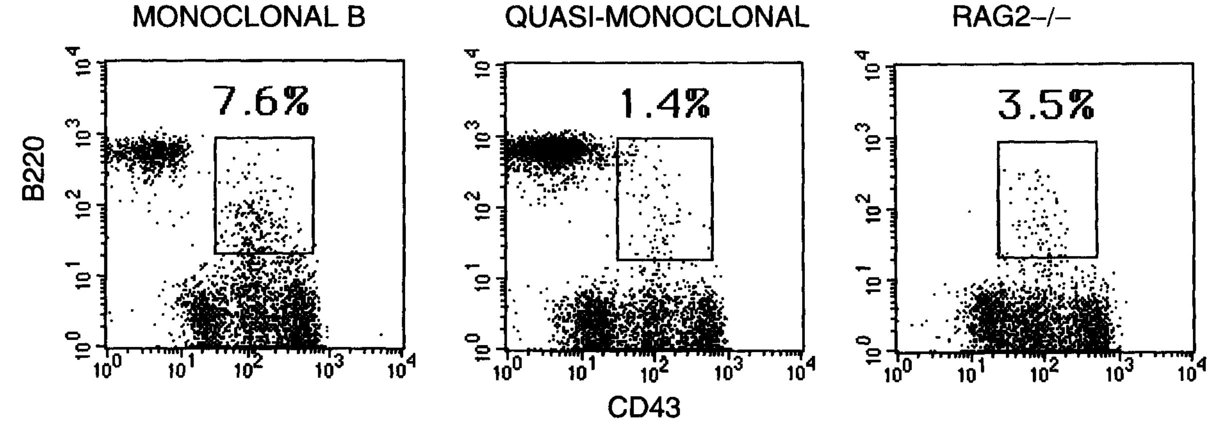

FIGURE 3 Flow-cytometric analysis of peripheral blood cells from MonoB, QM, and RAG $2^{-/-}$mice. *y*-axis; PE-conjugated anti-B220 monoclonal antibody. *x* axis: FITC-conjugated anti-CD43

out of the peripheral blood. Because T cells are important in promoting the germinal center reaction (Jacob and Kelsoe, 1992; MacLennan, 1994), the absence of T cells in MonoB mice might compromise the homing of lymphocytes that have bound antigen, thus leaving many of them circulating in the peripheral blood with masked λ and id epitopes. Because reacting MonoB PBL with the cognate hapten bound to a carrier abrogated the binding of the anti-id antibody (M. Madan, unpublished), the masked epitope notion is our working hypothesis.

Consistent with this hypothesis is the lack of cells with switched isotypes and the lack of circulating antibody other than IgM in MonoB mice, because switching also takes place in germinal centers. Interestingly, there are switched isotypes in a similar strain with another inserted $V_HDJ_H$, B1–8, and the same λ1 transgene on a RAG-2-deficient background (Lansford et al., 1998). As the only known significant difference between the two strains is the sequence of the inserted $V_HDJ_H$ gene segment and, as a consequence, the fine specificity of the BCR (White-Scharf and Imanishi-Kari, 1982; David et al., 1992), it is credible that the difference in switching behavior is due to antigen binding.

**Why Is There No Diversification by Transfer of Donor Sequences in MonoB Mice ?**

In the QM mouse and other gene-targeted mice (Chen et al., 1995; Taki et al., 1995; Cascalho et al, 1996; 1997), V-gene replacements are strongly selected and most new BCRs are generated by this mechanism. The selective forces must be at least as great in the MonoB mouse, which has an even smaller primary repertoire. Here we have shown that the primary repertoire of MonoB mice is not further diversified – by V-gene replacement, gene conversion, or any other process involving transfer of donor sequences – at a frequency detectable by current methods, even in a system with strong selective pressures. The interpretation of this result is complicated by the fact that the absence of RAG-2 expression in MonoB mice affects B cells in two ways – directly through the absence of V(D)J rearrangement and indirectly through the absence of T cells.

Whatever the nature of the cells lacking the idiotypic BCR of MonoB mice, any cells with V-gene replacement at the H-chain locus would be found in the id-negative fraction (Cascalho et al., 1997). Among 83 $V_HDJ_H$ segments sequenced from the id-negative population, there were no replacements. As the id-negative population used was 25% to 30%

of all B cells, we have effectively sampled about 300 cells, which means that at the 95% confidence level, there are less than 1% replacements. That is, the frequency of V-gene replacement is at least 100-fold lower than in the QM mouse. As it is not likely that the absence of T cells could result in such a large depression of the replacement frequency, the absence of V-gene replacement must be, at least in part, a direct consequence of the absence of V(D)J recombinase.

Gene conversion, another diversifying mechanism that is common in chickens and rabbits (Reynaud et al., 1987; Knight and Becker, 1990; David et al., 1992; Takeda et al., 1992), was also not observed. If it occurs at all in mouse germinal-center B cells, it is very rare (Ford et al., 1994). From our results, it is not clear whether its absence is due to dependence on RAG-2, T cells, or its rarity.

### MATERIALS AND METHODS

### Mice Strains and Breeding

The MonoB mouse was generated by breeding three strains: the QM mouse (Cascalho et al., 1996), a λ1 transgenic (Young et al., 1994), and a RAG-$2^{-/-}$ (Shinkai et al., 1992). After appropriate breeding, mice were screened by flow cytometry for the absence of T cells in peripheral blood lymphocytes (PBL) with an anti-CD3 antibody, a kind gift of Phillipe Poussier (Toronto), and for the presence of idiotype 17.2.25 (id) positive B cells with antibody R2.438.8, a kind gift of Thereza Imanishi-Kari (Tufts), who produced it; a detailed characterization of R2.438.8 has not yet been published. Expression of λ in PBL was also verified by immunofluorescence on cytospin slides or in flow cytometry with a goat anti-mouse λ polyclonal antibody (Pharmingen 01274D). In some breedings, the presence of the λ1 transgene and the 17.2.25 H-chain gene was detected by PCR analysis of tail DNA. For λ1, the primers and conditions are described in what follows in the cloning strategy; for H chain, see reference Cascalho et al. (1997). The genotype of each MonoB mouse was verified by staining peripheral blood lymphocytes (PBL) and PCR-based sequencing of the λ1 transgene.

### Cell Sorting and Flow Cytometry

Cells were analyzed with a Becton Dickinson (BD) FACScan or FACSCalibur and sorted with a FACStar or FACStarplus; data were analyzed with the BD Cell Quest program. PBL were prepared from total blood by hemolyzing erythrocytes with lysis buffer (8.2 g $NH_4Cl$, 1 g $KHCO_3$, 200 μl 0.5 M EDTA, pH 8.0, in 1 liter), washed, and stained by incubating them with antibodies for 30 min at 4°C before analysis and/or sorting. Dead cells were excluded by propidium iodide staining or with Via-Probe (Pharmingen 34321X 7-AAD). The following fluorescent antibodies were used for staining cells: phycoerythrin (PE) conjugated rat anti-B220 (Caltag RM2604), PE-conjugated mouse anti-B220 (mAb14.8, Pharmingen 01975B), PE-conjugated rat monoclonal anti-CD19 (mAb ID3, Pharmingen 09655B), PE-conjugated mouse monoclonal anti-$\mu^a$ (Pharmingen 05095B), monoclonal fluorescein isothiocyanate (FITC) coupled anti-17.2.25 $V_HDJ_H$ anti-idiotypic mAb R2.438.8, FITC-conjugated anti-λ (Pharmingen 01274D), which reacts with λ1 and λ2.

### LPS-Stimulated Spleen-Cell Cultures

Spleen-cell suspensions, prepared by standard methods, were placed in 24-well plates at 70,000 cell/well in RPMI, 10% FCS, and 10 μg/ml LPS. Six days later, wells were harvested, and clones were generated for sequencing as described in what follows for $V_HDJ_H$ and $C_\mu 1$–2 cDNA.

TABLE I Summary of MonoB H and L-Chain Gene Segments Sequenced

| *Cells*[a] | *Ig chain* | *Genomic or cDNA* | *Clones sequenced* | *Total bp sequenced* | *V-gene replacement* |
|---|---|---|---|---|---|
| Sorted B220$^+$ id$^-$ | | | | | |
| or $\mu^+$ id$^-$ PBL | H | cDNA | 45 | 12,720 | 0 |
| Sorted $\mu^+$ id$^-$ PBL (LPS-activated spleen) | H(H) | Genomic (cDNA) | 38 (16) | 13,300 (4,165) | 0 (0) |
| Totals | H | | 99 | 30,185 | 0 |
| Sorted $\mu^+$ id$^-$ PBL | | | | | |
| or B220$^+$ id$^-$ PBL | λ1 | cDNA | 21 | 7,670 | 0 |
| Sorted B220$^+$ λ$^-$PBL | λ1 | cDNA | 20 | 7,551 | 0 |
| Totals | λ1 | | 41 | 15,221 | 0 |

id$^-$: cells not stained by anti-idiotype 17.2.25 monoclonal antibody (see Fig. 1). PBL: peripheral blood lymphocytes.

### Preparation of cDNA and DNA and amplification by PCR

An Oligotex direct mRNA kit (Quiagen 72012) was used to prepare mRNA, which was reverse transcribed with Superscript (Gibco-BRL 180–014) to obtain cDNA. The synthesis was primed with oligo $d(T)_{12-18}$ (Gibco-BRL 18418-012). For DNA, a published direct-cell lysate procedure was used (Pennycook et al., 1997). For both DNA and cDNA, a partial $V_HDJ_H$ was amplified by PCR with 5 pmol $V_H$ 5′ primer AGGTc/ga/cAa/gCTGCAGc/gAGTCa/tGG (Orlandi et al., 1989) and $J_H4$ 3′ primer GAGGAGACGGTGACTGAGGTTCCTTG ; λ 1 cDNA was amplified with $V_\lambda 1$ 5 primer GGAATTCCTGCACTCACCACATCACCTGG and $C_\lambda 1$ 3′ primer GTGGATCCTACCTTCCAGTCCACTGTCACC; $C_\mu$ cDNA was amplified with $C_\mu 1$ 5′ primer GAGTCAGTCCTTCCCAAATG and $C_\mu 2$ 3′ primer CTCGATGGTCACCGGATCTG. Reactions were done in 50 µl total volume with 2.5 U Taq polymerase (BMB 1146173), 200 µM of dNTPs 1X PCR buffer, with 1.8 mM $MgCl_2$ for the $V_HDJ_H$segment, and 1.5 mM $MgCl_2$ for the λ1 gene and $C_\mu$gene segment. For the $V_HDJ_H$ and $C_\mu$gene segments, the program ran 30 cycles, each 30 sec, 94°C; 1 min, 60°C; 2 min, 72°C; and for λ 40 cycles, each 1 min, 94°C; 1 min, 59°C; and 2 min, 72°C. The last cycle included a prolonged extension step (10 min). The PCR fragments were cloned into pCR 2.1 (Invitrogen K2000–01).

### Sequencing

Double-stranded DNA was prepared and clones were screened for the presence of the insert by digestion with EcoRI. Positive clones were sequenced with the Sequenase kit (USB 70770) and the T7 Sequencing Kit (Pharmacia Biotech 27–1682–01) and universal or reverse primers.

### *Acknowledgements*

We are indebted to Claude Cantin for flow cytometry and cell sorting and to Charley Steinberg for help with the manuscript. This work was supported by NIH grant R01 AI41570, by the National Cancer Institute of Canada Terry Fox Marathon of Hope, by the Medical Research Council of Canada (MRC), by an Engalitcheff Award of the Arthritis Foundation, by the Leukemia Research Fund of Canada, by funds from the Markey Trust, by a Howard Hughes Transgenic Mouse Grant, and by a grant from the Junta Nacional de Investigação Científica e Tecnológica – Praxis XXI-BD 3763/94 to MC. DM is the recipient of a MRC studentship; GEW is an MRC Scientist.

### *References*

Beck-Engeser, G., Jäck, H.-M., and Wabl, M. (1987). Allelic inclusion in a pre-B-cell line that generates immunoglobulin heavy chain genes in vitro. *Proc. Natl. Acad. Sci. USA* **84:** 1060–1064.

Cascalho, M., Ma, A., Lee, S., Masat, L., and Wabl, M. (1996). A quasi-monoclonal mouse. *Science* **272:** 1649–1652.

Cascalho, M., Wong, J. and Wabl M. (1997). $V_H$ gene replacement in hyperselected B cells of the quasi-monoclonal mouse. *J. Immunol.* **159:** 5795–5801.

Chen, C., Nagy, Z., Prak, E.L., and Weigert, M. (1995). Immunoglobulin heavy chain gene replacement: A mechanism of receptor editing. *Immunity* **3**: 747–755.

Chen, J., Trounstine, M., Alt, F. W., Young, F., Kurahara, C., Loring, J.F., and Huszar, D. (1993a). Immunoglobulin gene rearrangement in B cell deficient mice generated by targeted deletion of the JH locus. *Int. Immunol.* **5**: 647–856.

Chen, J., Trounstine, M., Kurahara, C., Young, F., Kuo, C. C., Xu, Y., Loring, J. F., Alt, F. W., and Huszar, D. (1993b). B cell development in mice that lack one or both immunoglobulin kappa light chain genes. *EMBO J.* **12**:821–830.

Covey, L. R., Ferrier, P., and Alt, F. W. (1990). VH to VHDJH rearrangement is mediated by the internal VH heptamer. *Int. Immunol.* **2**:579–583.

David, V., Folk, N. L. and Maizels, N. (1992). Germline variable regions that match hypermutated sequences in genes encoding murine anti-hapten antibodies. *Genetics* **132**: 799–811.

Fanning, L., Bertrand, F. E., Steinberg, C., and Wu, G. E. (1998). Molecular mechanisms involved in receptor editing at the Ig heavy chain locus. *Int. Immunol.* **10**: 241–246.

Ford, J. E., McHeyzer-Williams, M. G., and Lieber, M. R. (1994). Analysis of individual immunoglobulin lambda light chain genes amplified from single cells is inconsistent with variable region gene conversion in germinal-center B cell somatic mutation. *Eur. J. Immunol.* **24**: 1816–1822.

Jacob, J., and Kelsoe, G. (1992). In situ studies of the primary immune response to (4-hydroxy-3-nitrophenyl)acetyl. II. A common clonal origin for periarteriolar lymphoid sheath-associated foci and germinal centers. *J. Exp. Med.* **176**: 679–687.

Kleinfield, R., Hardy, R. R., Tarlington, D., Dangl, J., Herzenberg, L. A., and Weigert M. (1986). Recombination between an expressed immunoglobulin heavy-chain gene and a germline variable gene segment in a Ly1+ B-cell lymphoma. *Nature* **322**: 843–846.

Kleinfield, R. W., and Weigert, M. G. (1989). Analysis of $V_H$ gene replacement events in a B cell lymphoma. *J. Immunol.* **142**: 4475–4482.

Knight, K. L., and Becker, R. S. (1990). Molecular basis of the allelic inheritance of rabbit immunoglobulin VH allotypes: Implications for the generation of antibody diversity. *Cell* **60**:963–970.

Lansford, R., Manis, J. P., Sonoda, E., Rajewsky, K., and Alt, F. W. (1998). Ig heavy chain class switching in Rag-deficient mice. *Int. Immunol.* **10**: 325–332.

MacLennan, I. C. (1994). Germinal centers. *Annu. Rev. Immunol.* **12**: 117–139.

Orlandi, R., Gussow, D. H., Jones, P. T. and Winter, G. (1989). Cloning immunoglobulin variable domains for expression by the polymerase chain reaction. *Proc. Natl. Acad.Sci. USA* **86**: 3833–3837.

Pennycook, J. L. M. H., Marshall, A. J., and Wu, G. E. (1997). PCR assays for endogenous Ig gene rearrangement. In *Immunology Methods Manual,* Lefkovits I., Ed. (London: Academic Press). pp. 239.

Reth, M., Gehrmann, P., Petrac, E., and Wiese, P. (1986). A novel $V_H$ to $V_HDJ_H$ joining mechanism in heavy-chain-negative (null) pre-B cells results in heavy-chain production. *Nature* **322**: 840–842.

Reynaud, C. A., Anquez, V., Grimal, H., and Weill, J. C. (1987). A hyperconversion mechanism generates the chicken light chain preimmune repertoire. *Cell* **48**: 379–388.

Shinkai, Y., Rathbun, G., Lam, K.P., Oltz, E., Stewart, V., Mendelsohn, M., Charron, J., Datta, M., Young, F., Stall, A.M., and Alt, F. (1992) RAG-2 deficient mice lack mature lymphocytes owing to inability to initiate V(D)J recombination. *Cell* **68**: 855–867.

Shirasawa, T., Miyazoe, I., Hagiwara, S., Kimoto, H., Shigemoto, K., Taniguchi, M., and Takemori, T. (1992). Heavy chain variable ($V_H$) region diversity generated by $V_H$ gene replacement in the progeny of a single precursor cell transformed with a temperature-sensitive mutant of Abelson murine leukemia virus. *J. Exp. Med.* **176**: 1209–1214.

Steenbergen E. J., Verhagen, O. J ., van Leeuwen, E. F., von dem Borne, A. E., and van der Schoot, C. E. (1993). Distinct ongoing Ig heavy chain rearrangement processes in childhood B-precursor acute lymphoblastic leukemia. *Blood* **82**: 581–589.

Takeda, S., Masteller, E. L., Thompson, C. B. and Buerstedde, J. M. (1992). RAG-2 expression is not essential for chicken immunoglobulin gene conversion. *Proc. Natl. Acad. Sci. USA* **89**:4023–4027.

Taki, S., Schwenk, F., and Rajewsky, K. (1995). Rearrangement of upstream DH and VH genes to a rearranged immunoglobulin variable region gene inserted into the DQ52-JH region of the immunoglobulin heavy chain locus. *Eur. J. Immunol.* **25**: 1888–1896.

Usuda, S., Takemori, T., Matsuoka, M., Shirasawa, T., Yoshida, K., Mori, A., Ishizaka, K., and Sakano, H. (1992). Immunoglobulin V gene replacement is caused by the intramolecular DNA deletion mechanism. *EMBO J.* **11**: 611–618.

White-Scharf, M. E., and Imanishi-Kari, T. (1982). Cross-reactivity of NPa and NPb idiotypic responses of BALB/c and C57BL/6 mice to (4-hydroxy-3-nitrophenyl)acetyl (NP). *Eur. J. Immunol.* **12**: 935–942.

Young, F., Ardman, B., Shinkai, Y., Lansford, R., Blackwell, T. K., Mendelsohn, M., Rolink, A., Melchers, F., and Alt, F. (1994). Influence of immunoglobulin heavy- and light-chain expression on B-cell differentiation. *Genes Devel.* **8**: 1043–1057.

